# Physiological Benefits of Being Small in a Changing World: Responses of Coho Salmon (*Oncorhynchus kisutch*) to an Acute Thermal Challenge and a Simulated Capture Event

**DOI:** 10.1371/journal.pone.0039079

**Published:** 2012-06-13

**Authors:** Timothy D. Clark, Michael R. Donaldson, Sebastian Pieperhoff, S. Matthew Drenner, Andrew Lotto, Steven J. Cooke, Scott G. Hinch, David A. Patterson, Anthony P. Farrell

**Affiliations:** 1 Department of Forest Sciences, University of British Columbia, Vancouver, British Columbia, Canada; 2 Centre for Cardiovascular Science, Queen's Medical Research Institute, University of Edinburgh, Edinburgh, Scotland, United Kingdom; 3 Fish Ecology and Conservation Physiology Laboratory, Department of Biology and Institute of Environmental Science, Carleton University, Ottawa, Ontario, Canada; 4 Fisheries and Oceans Canada, Cooperative Resource Management Institute, School of Resource and Environmental Management, Simon Fraser University, Burnaby, British Columbia, Canada; 5 Department of Zoology, University of British Columbia, Vancouver, British Columbia, Canada; Institute of Marine Research, Norway

## Abstract

Evidence is building to suggest that both chronic and acute warm temperature exposure, as well as other anthropogenic perturbations, may select for small adult fish within a species. To shed light on this phenomenon, we investigated physiological and anatomical attributes associated with size-specific responses to an acute thermal challenge and a fisheries capture simulation (exercise+air exposure) in maturing male coho salmon (*Oncorhynchus kisutch*). Full-size females were included for a sex-specific comparison. A size-specific response in haematology to an acute thermal challenge (from 7 to 20°C at 3°C h^−1^) was apparent only for plasma potassium, whereby full-size males exhibited a significant increase in comparison with smaller males (‘jacks’). Full-size females exhibited an elevated blood stress response in comparison with full-size males. Metabolic recovery following exhaustive exercise at 7°C was size-specific, with jacks regaining resting levels of metabolism at 9.3±0.5 h post-exercise in comparison with 12.3±0.4 h for full-size fish of both sexes. Excess post-exercise oxygen consumption scaled with body mass in male fish with an exponent of *b* = 1.20±0.08. Jacks appeared to regain osmoregulatory homeostasis faster than full-size males, and they had higher ventilation rates at 1 h post-exercise. Peak metabolic rate during post-exercise recovery scaled with body mass with an exponent of *b*∼1, suggesting that the slower metabolic recovery in large fish was not due to limitations in diffusive or convective oxygen transport, but that large fish simply accumulated a greater ‘oxygen debt’ that took longer to pay back at the size-independent peak metabolic rate of ∼6 mg min^−1^ kg^−1^. Post-exercise recovery of plasma testosterone was faster in jacks compared with full-size males, suggesting less impairment of the maturation trajectory of smaller fish. Supporting previous studies, these findings suggest that environmental change and non-lethal fisheries interactions have the potential to select for small individuals within fish populations over time.

## Introduction

Evidence exists to suggest that both chronic and acute warm temperature exposure may benefit small animal species and small adult individuals within a species [1], [2], [3], [4], [5]. A negative correlation between the thermal environment and body size was first proposed over 160 years ago when Bergmann [6] documented this phenomenon for endothermic vertebrates over a large spatial scale. Since then, the topic has been heavily debated while scientists have strived to confirm the phenomenon, provide a mechanism, and extend the concept to ectothermic vertebrates [1], [3], [7], [8], [9].

For fishes, climate change and variability are altering species distributions as populations respond to warming waters (e.g., [10]), and there is some correlative evidence to suggest intraspecific selection based on thermal interactions with body size [1], [11]. A study of Chinook salmon (*Oncorhynchus tshawytscha*) suggested that small individuals may be more tolerant of an acute thermal challenge than larger conspecifics [5], which has important ecological implications for all Pacific salmonids since they can experience abrupt and extreme temperature challenges during their freshwater migrations. Since the majority of information on the interaction between temperature tolerance and animal size stems from descriptive observations rather than controlled studies, contention surrounds the physiological mechanisms underlying the observed trends (e.g., see [8]).

An additional challenge faced by many fish species is the increasing likelihood of interaction with commercial or recreational fisheries. Fisheries capture may result in direct mortality, although fish commonly escape following contact with fishing gear, or they are intentionally released as bycatch or through catch-and-release fisheries. Fishes can encounter similar stressors under natural conditions, such as during non-lethal interactions with animal predators (e.g., seals, birds, bears). Released or escaped fish must physiologically recover from the encounter or risk mortality [12]. Since many retention fisheries are regulated based on fish size, it is of importance to understand whether body size plays a role in post-release recovery and survival, yet no previous study has investigated this possibility.

Given the sparse dataset concerning these issues, the present study sought to explore a range of physiological and anatomical attributes that might benefit small individuals within a species when exposed to an acute thermal challenge or a simulated fisheries/predator encounter. Attention was focussed on the oxygen transport system and blood chemistry, as these have been previously implicated in determining the tolerance of fishes to various environmental challenges [4], [13], [14]. Coho salmon (*Oncorhynchus kisutch*) was chosen as the model species for several reasons: (1) freshwater and marine ecosystems utilised by coho salmon have warmed, and fish now have a greater probability of acutely encountering higher river temperatures than at any point since records began in the 1940s [15], [16], [17], (2) coho salmon can experience marked and rapid thermal increments throughout their lifecycle [18], [19], (3) significant intergenerational declines in adult body mass have occurred over time in wild populations of this species [20], [21], (4) coho salmon is a target species for fisheries and large predators including bears and seals, (5) mandatory release exists for some populations of imperilled coho salmon incidentally caught as bycatch in other fisheries ([22] and references within), and (6) the species displays a diverse range in life history strategies that leads to significant variation in adult body size, which provides a novel opportunity to examine the intraspecific effects of body size on fish at the same stage of sexual maturation.

## Materials and Methods

### Animals

Mature coho salmon (*Oncorhynchus kisutch*) were dip-netted throughout October and November in 2009 and 2010 as they completed their 140 km upstream migration from the Pacific Ocean to their natal rearing location at the Chehalis River Hatchery (British Columbia, Canada), where they had been released previously as one year old fish. Following their first year in freshwater, most individuals spend 1.5–2.5 years foraging in the marine environment prior to returning to their natal freshwater rearing location as 2.5–3.5 year-olds (∼1.5–6 kg; 53–83 cm fork length [FL]), yet some individuals (primarily males) mature and return to the rearing location after only 6 months in the ocean and at a much smaller body size (0.20–0.65 kg; 26–39 cm FL). Full-size males (2.5–3.5 years old) and precocious males (1.5 years old; hereafter referred to as ‘jacks’) were used in the present study to investigate the effects of body size (fish age was accurately estimated from fish size, as determined by scale analyses; Steve Latham, Pacific Salmon Commission, BC, Canada). Full-size females were also used to examine for sex-specific differences in full-size fish. Precocious females can occur but are extremely rare, and none were encountered throughout the two years in which the present study was conducted. While it is generally unavoidable to have age and size covarying when examining intraspecific body mass (*M*_b_) scaling in fishes, we believe that the male coho salmon model used here is better than most because the fish are not feeding/growing and they are at similar stages of reproductive maturity. All experiments were conducted with the approval of the Animal Care Committee of the University of British Columbia, in accordance with the Canadian Council on Animal Care.

### Thermal challenge

This protocol was designed to investigate whether jacks experienced less blood physiological disturbance (i.e., deviations from control values) than full-size fish when exposed to an acute thermal challenge. Coho salmon (15–16 fish per trial) were placed individually into opaque holding tubes equipped with plastic mesh at each end to allow good flow-through of water. The tubes were either 90×22 cm (length×diameter) for full-size fish or 70×12 cm for jacks. The tubes were evenly (or near-evenly) distributed between two enclosures, both containing flow-through river water at ambient temperature (∼7°C). The tubes were arranged side-by-side and also stacked 2–3 high, such that all fish within each enclosure were housed within a space of ∼1.5 m^3^.

All fish were given 20 h to recover from handling before the group in one enclosure (warm) was exposed to an acute thermal challenge while the group in the second enclosure (control) remained at ambient temperature and acted as a control. A gas-powered water heating system was used to increase the temperature of the warm enclosure by 3°C h^−1^ from 7 to 20°C, after which the fish were maintained at 20°C for a further 2 h. At this point, all fish from the control enclosure (7°C) and warm enclosure (20°C) were individually removed from the holding tubes, sacrificed by cerebral concussion, and sampled for blood (∼3 ml) from the caudal vasculature using lithium-heparinised vacutainers. Extreme care was taken to avoid disturbing each individual until <30 s before it was removed and sampled for blood, as this is known to provide a blood sample that is uninfluenced by the handling event [23]. An upper temperature of 20°C was chosen on the basis of previous data [18], [19], [24], since this was anticipated to maximally test the aerobic capacity of coho salmon without surpassing upper thermal limits.

Three identical trials were conducted on separate days, giving total *M*_b_ ranges and sample sizes (at 7 and 20°C, respectively) of: full-size males, *M*_b_ = 1.39–4.22 kg, N = 9 and *M*_b_ = 1.39–3.71 kg, N = 7; full-size females, *M*_b_ = 2.06–3.35 kg, N = 9 and *M*_b_ = 1.31–3.93 kg, N = 8; jacks, *M*_b_ = 0.20–0.51 kg, N = 7 and *M*_b_ = 0.22–0.60 kg, N = 7.

### Recovery from exhaustive exercise

The exhaustive exercise protocol was identical to that used previously [25], and was designed to simulate the exercise stress associated with fisheries capture (e.g., angling catch-and-release) and the period of air exposure that typically follows (e.g., for hook removal, photography, etc). Briefly, individual fish were placed into a ring-shaped tank (outer diameter 150 cm, inner diameter 50 cm, water depth 40 cm) and manually chased for 3 min, after which they were exposed to air in a dip-net for 1 min before being placed into a recovery box (L×W×D = 90×50×50 cm; water depth 30 cm) supplied with flow-through river water (50 l min^−1^; ∼7°C). The chasing protocol rendered all individuals unable to continue burst swimming after about 2 min. Sampling took place at 1, 2, 4, 8 or 19 h post-treatment, when individual fish were rapidly removed from the recovery box, sacrificed by cerebral concussion and sampled for blood by caudal puncture as described above. Full-size fish (both sexes) and jacks were examined. Ten recovery boxes were used in each trial, and trials were repeated to ensure appropriate sample sizes in each size-grouping and sex-grouping, and at each post-treatment recovery period (sample sizes in figures). Ventilation rate was measured in a subsample of fish (7 full-size males, 3 full-size females, 10 jacks) at 1 h post-exercise through a small viewing slit in the lid of each recovery box.

Additional fish (10 full-size males, 12 full-size females, 11 jacks) underwent the same treatment protocol as above but were placed individually and immediately into static respirometers (each 138 l) rather than recovery boxes during the post-treatment recovery period. This group of fish was not sampled for blood, but rather remained in the respirometers for 19 h while the rate of oxygen consumption (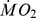) was recorded throughout the entire recovery period for 15 min every hour. The respirometers and techniques of measuring 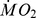 were identical to those used previously [13], except that water temperature remained at 7°C and the water volume of the respirometers was reduced using plastic-coated concrete blocks when measuring 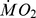 of jacks. Minimum and peak oxygen consumption rates (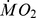
_min_ and 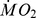
_peak_, respectively) were taken as the minimum and maximum values obtained for each individual throughout the 19 h recovery period, with the former always occurring after the latter.

Another group of full-size fish (3 males, 3 females) was surgically implanted with biologgers and given 6 d to recover before undergoing the exhaustive exercise protocol described above. Jacks were not used as they were too small to receive a biologger. Surgical methods were identical to those used previously [26], [27]. The biologgers took 10-s samples of the electrocardiogram (ECG) at 200 Hz every 10 min (details in [26]), which were subsequently used to calculate heart rate using LabChart software (ADInstruments, Sydney, Australia). This group of fish was not sampled for blood, but rather remained in the recovery boxes for 17 h so the biologgers could record the uninterrupted recovery period.

### Blood analyses and post-mortem measurements

Blood samples were immediately placed into an ice slurry and processed within 20 min. Haematocrit (Hct) of whole blood was measured using micro-capillary tubes spun at 10,000×*g* for 7 min. Remaining blood was spun at 7,000×*g* for 7 min and then the plasma was collected in cryogenic vials and stored in liquid nitrogen prior to being transferred to a −80°C freezer for subsequent analyses.

Single plasma measurements were made of lactate and glucose, with an internal calibration performed every five samples (YSI 2300 stat plus analyser; www.ysilifesciences.com). Plasma measurements were made in duplicate of cortisol (Neogen ELISA with Molecular Devices Spectramax 240 pc plate reader, Lexington, Kentucky, U.S.A.), osmolality (Advanced Instruments 3320 freezing point osmometer), chloride (Haake Buchler digital chloridometer), sodium and potassium (Cole-Parmer, model 410 single channel flame photometer) (see [28] for further details). The hormones testosterone and 17β-estradiol (the latter measured for females only) were assayed in duplicate after appropriate dilution and ether extraction (Neogen ELISA, Lexington, Kentucky, U.S.A.).

Selected fish underwent post-mortem dissection to measure organ weights. The gonads, spleen, liver and ventricle were removed and weighed following removal of excess blood. For some fish (including additional fish to those used in the above experiments), an incision was made around the perimeter of the opercular cavity and the entire gill basket was removed, rinsed, blotted and weighed. For six fish (3 full-size males, 3 jacks), a section of gill was removed immediately after death (first gill arch, left hand side) and fixed in 10% acetate-buffered formalin. The gill samples were subsequently dehydrated in an ethanol series and embedded in paraffin wax, before being sectioned at 5–8 µm and stained with Masson's trichrome. Images were taken for histological comparisons using an Olympus Provis AX70 microscope (Tokyo, Japan) in combination with a AxioCam HRc cooled CCD camera (Carl Zeiss, Jena, Germany). AxioVision software (Carl Zeiss, Jena, Germany) was used to measure the following variables. Blood-water diffusion distance, maximum erythrocyte diameter (targeting intact, uniform cells), and gill lamellae diameter were measured in images taken at 40×magnification. Gill lamellae length and interlamellar distance were measured in images taken at 10×magnification, while gill filament diameter was measured in images taken at 4×magnification (full-size fish) or 10×magnification (jacks). Ten measurements of each parameter were taken at random for each fish in areas where the gill filaments were least variable in diameter, distal from the gill arch.

### Statistics

Statistical tests were performed in SPSS (Build 16.0, SPSS Inc., Chicago, IL, USA) and SigmaStat (Build 3.01.0, Systat Software Inc., www.systat.com) using Bonferroni correction where necessary to account for multiple comparisons. All blood data for between group comparisons were log-transformed prior to statistical analyses to satisfy tests for normality. Statistical differences resulting from the thermal challenge were assessed using two-way ANOVA with group (full-size male, full-size female, jack) and temperature as factors, including the interaction term group*temperature. Two-way repeated measures ANOVA was used to investigate differences in 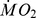 between groups during recovery from the exhaustive exercise protocol, and one-way ANOVA was used to investigate differences in blood variables between groups at each time point. Two parameter power regressions were used to examine body mass scaling of measured variables in male fish. Gill histological measurements were compared using two-way repeated measures ANOVA with group and measurement number (1 through 10) as factors.

## Results

### Thermal challenge

**Figure 1:**
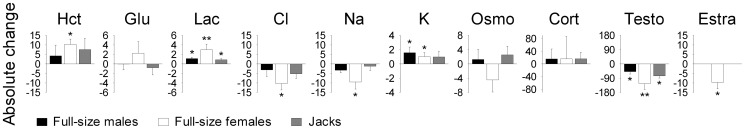
Changes in blood parameters of coho salmon (*O. kisutch*) upon exposure to an acute temperature increase from 7 to 20°C. Full-size males, full-size females, and jacks are presented (N given in *Materials and methods*). The blood parameters and their corresponding units are: Hct (haematocrit, %), Glu (glucose, mmol l^−1^), Lac (lactate, mmol l^−1^), Cl (chloride, mmol l^−1^), Na (sodium, mmol l^−1^), K (potassium, mmol l^−1^), Osmo (osmolality, mOsm kg^−1^), Cort (cortisol, ng ml^−1^), Testo (testosterone, ng ml^−1^), and Estra (17β-estradiol, ng ml^−1^). All variables were measured in blood plasma, except Hct which was measured in whole blood. Two-way ANOVA was used for statistical comparisons (see *Statistics*). * significantly different from zero (i.e., significantly different from the control group held at 7°C) at the level of P<0.05; ** significantly different from zero at the level of P<0.001. Full-size males and jacks did not differ in any blood parameter within the 7°C group or within the 20°C group. The only difference between these two groups was that full-size males had a significant increase in plasma potassium from 7 to 20°C, whereas jacks did not.

Increasing water temperature to 20°C had a significant effect on the blood properties of all groups of fish in comparison with the respective control groups held at 7°C (two-way ANOVA; Fig. 1). While the qualitative trends were similar for full-size fish (both sexes) and jacks, the thermally-induced changes tended to be magnified for full-size females (though the group*temperature interaction in the two-way ANOVA was not significant for any blood variable). Specifically, Hct, plasma lactate and potassium all increased significantly with temperature, while plasma chloride, sodium, testosterone and 17β-estradiol decreased. Within males, the only variable to differ significantly with body size was plasma potassium, which increased significantly with temperature in full-size males but not in jacks (Fig. 1). The only fish that did not survive the thermal challenge was a full-size mature male, which was the largest male in the trial (3.7 kg). Blood was sampled from this fish following death but the data were not included in Fig. 1. Notably, plasma lactate (17.0 mmol l^−1^) and potassium (13.3 mmol l^−1^) were higher in this fish than in any other individual, while plasma testosterone (2.2 ng ml^−1^) was the lowest of all fish.

### Recovery from exhaustive exercise

**Figure 2:**
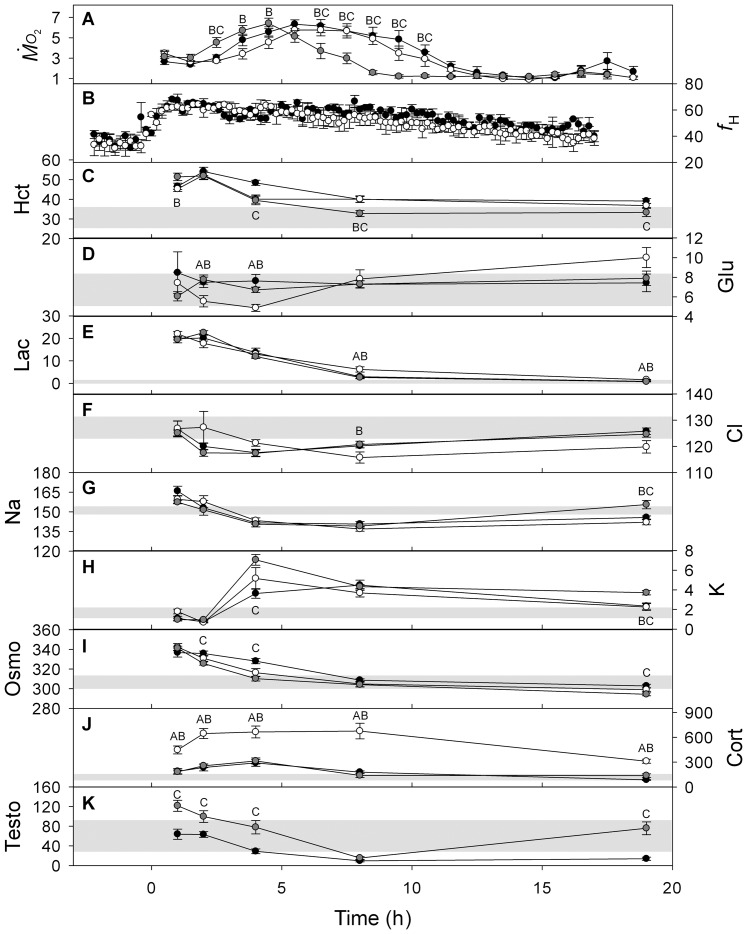
Responses in cardiorespiratory variables and blood properties of coho salmon (*O. kisutch*) following a standard treatment (3 min exhaustive exercise plus 1 min air exposure) at 7°C. The treatment concluded, and recovery commenced, at Time = 0 h. Full-size males (black), full-size females (white), and jacks (grey) are presented. Rates of oxygen consumption (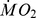) were measured using respirometry (N = 10 full-size males, N = 12 full-size females, N = 11 jacks), and heart rates (*f*_H_) were measured using surgically implanted biologgers (N = 3 full-size males, N = 3 full-size females). Abbreviations and units for the blood variables are given in Fig. 1 (for blood variables, N = 10–14 for each group at each time point, except at 4 h where N = 5–10 for each group). Letters near data points denote significant differences (P<0.05) within a given time interval between (A) full-size males and full-size females, (B) full-size females and jacks, and (C) full-size males and jacks. Shaded horizontal bars represent mean±SD reference values for full-size male *O. kisutch* taken from Clark et al. [23] (raw data from caudal sample in table II).

The exercise and air exposure protocol caused complete exhaustion in all groups of fish (no capacity to continue burst swimming) and a significant increase in 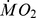 (Fig. 2A). The 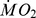 recovery profile following the exercise protocol was similar for full-size males and females, where 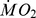 began to increase after ∼3 h, peaked between 6–8 h, and then gradually declined to resting levels by 12.3±0.4 h post-exercise. While jacks achieved the same minimum and peak levels of 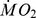 as full-size fish (i.e., ∼1 and ∼6 mg min^−1^ kg^−1^, respectively [use of isometric scaling justified below]), the initial increase in 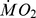 began about 1 h earlier than for full-size fish, and metabolic recovery was complete at 9.3±0.5 h rather than 12.3 h. Ventilation rate, measured in a subsample of fish at 1 h post-exercise, was higher in jacks (75±3 beats min^−1^; 0.40±0.03 kg) than in full-size fish (62±2 beats min^−1^; 3.99±0.28 kg; t-test P = 0.002).

The exercise protocol caused an abrupt increase in heart rate of full-size fish (both sexes), which progressively decreased to resting levels over a longer time period than 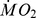 (i.e., ∼17 h; Fig. 2B). There were no sex-specific differences in heart rate, despite the potential for the elevated plasma cortisol of females to have a positive chronotropic effect (e.g., [29]). It was not possible to determine the role of heart rate in the size-specific differences documented for 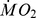 because jacks were too small to be implanted with biologgers.

Blood variables clearly illustrated the stress response linked with the exhaustive exercise protocol, such as increases in Hct, plasma lactate and cortisol, and disruption to plasma ion concentrations (Fig. 2). Plasma potassium and osmolality differed between full-size males and jacks at certain points throughout the recovery period, suggesting that these variables may play some role in the size-specific difference in metabolic recovery (Fig. 2H, 2I). Additionally, the delayed increase in plasma potassium was similar to that of 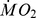 (i.e., 2–4 h post-exercise). However, there was no size-specific difference in plasma potassium at 8 h post-exercise, contrary to expectations based on the marked size-specific difference in 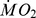 at that time point (Fig. 2A, 2H). One of the most obvious size-specific haematological findings was the higher plasma testosterone concentration in jacks compared with full-size males, and the ability of jacks to regain high levels of testosterone at 19 h post-exercise while testosterone of full-size males remained depressed (Fig. 2K).

### Body mass scaling

**Figure 3:**
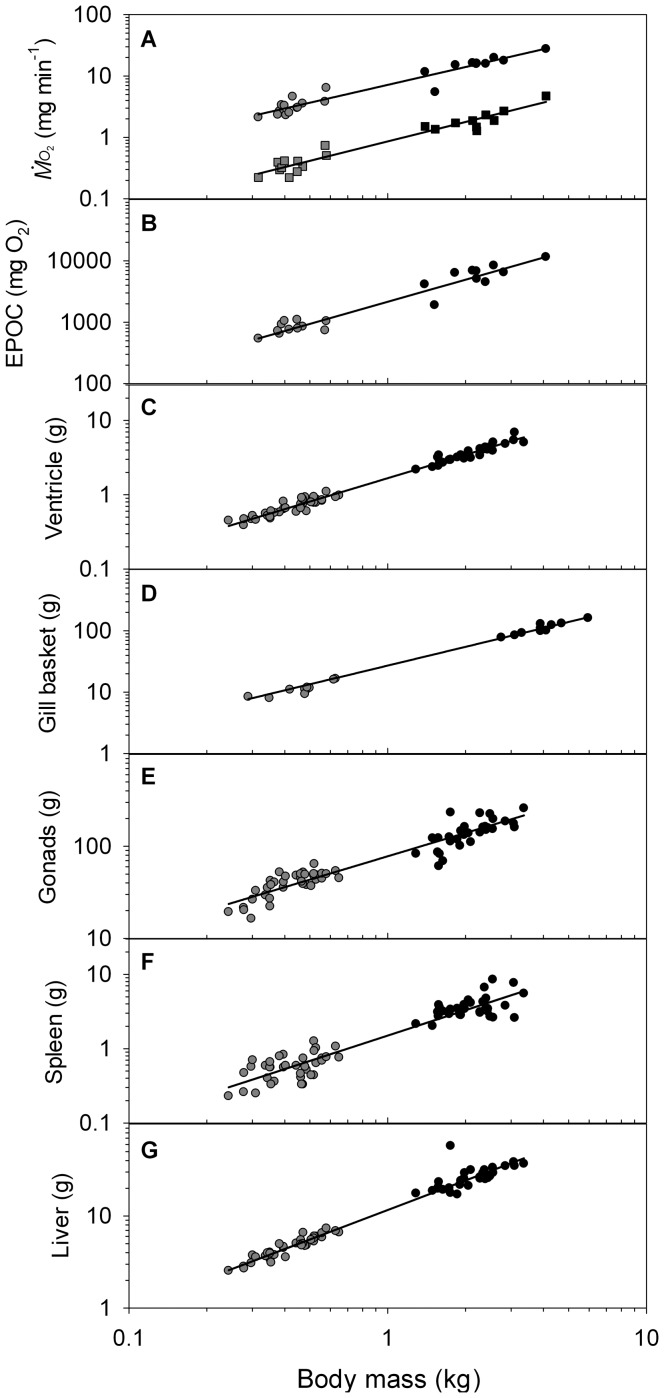
Relationships between body mass (*M*_b_) and metabolic and anatomical parameters of coho salmon (*O. kisutch*). (A) minimum (squares) and peak (circles) oxygen consumption rate (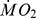
_min_ and 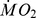
_peak_, respectively), (B) excess post-exercise oxygen consumption (EPOC), and (C–G) anatomical parameters. Only data for full-size males (black) and jacks (grey) are presented to remove variability associated with sex-specific differences. Regression lines (with standard errors in parentheses) are described by: (A) 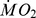
_min_ = 0.861 (0.047) • *M*_b_^1.053 (0.063)^ (R^2^ = 0.935, P<0.001), 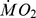
_peak_ = 7.103 (0.339) • *M*_b_^0.968 (0.056)^ (R^2^ = 0.935, P<0.001); (B) EPOC = 2154.6 (136.9) • *M*_b_^1.196 (0.075)^ (R^2^ = 0.927, P<0.001); (C) ventricle mass = 1.662 (0.022) • *M*_b_^1.045 (0.016)^ (R^2^ = 0.985, P<0.001); (D) gill basket mass = 25.519 (0.589) • *M*_b_^1.061 (0.020)^ (R^2^ = 0.993, P<0.001); (E) gonad mass = 76.906 (2.113) • *M*_b_^0.824 (0.033)^ (R^2^ = 0.907, P<0.001); (F) spleen mass = 1.503 (0.060) • *M*_b_^1.134 (0.047)^ (R^2^ = 0.897, P<0.001); (G) liver mass = 11.625 (0.236) • *M*_b_^1.066 (0.024)^ (R^2^ = 0.968, P<0.001).

Physiological and morphological data were compared across all males, which spanned over an order of magnitude in body mass (Fig. 3). The mass scaling exponents (*b*) were 1.05±0.06 for minimum 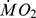 (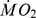
_min_; taken as the lowest 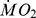 recorded post-recovery) and 0.97±0.06 for peak 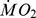 (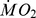
_peak_; taken as the highest 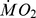 recorded during the recovery period). Thus, the factorial aerobic scope (calculated as 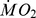
_peak_/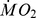
_min_) did not differ substantially with increasing body mass. Excess post-exercise oxygen consumption (EPOC; calculated for each individual as the total amount of oxygen consumed above 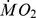
_min_ throughout the recovery period) was disproportionately higher in full-size males than in jacks (*b* = 1.20±0.08; Fig. 3B), which reflected the longer duration of metabolic recovery in full-size males (*b*∼0.16; see above) rather than any size-specific differences in mass-specific 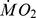
_min_ or 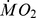
_peak_.

**Figure 4:**
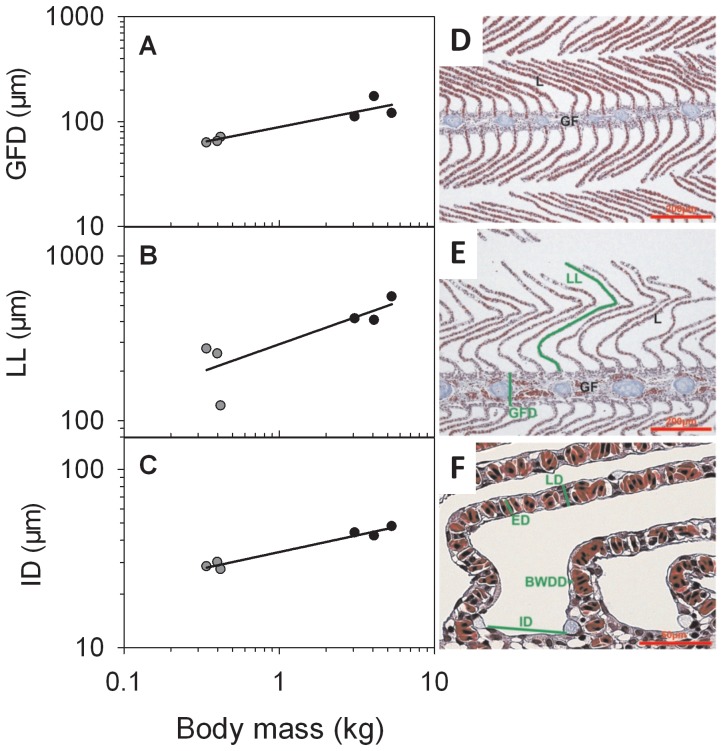
Effects of body mass (*M*_b_) on gill morphology of coho salmon (*O. kisutch*). Relationships between *M*_b_ and each of (A) gill filament diameter (GFD), (B) lamellae length (LL), and (C) interlamellar distance (ID) for jacks (grey symbols) and full-size fish (black symbols). Regression lines (with standard errors in parentheses) are described by: (A) GFD = 87.667 (6.180) • *M*_b_^0.293 (0.058)^ (R^2^ = 0.863; P = 0.007); (B) LL = 284.539 (40.478) • *M*_b_^0.336 (0.118)^ (R^2^ = 0.672; P = 0.046); (C) ID = 34.355 (0.735) • *M*_b_^0.187 (0.018)^ (R^2^ = 0.966; P<0.001). Histological preparations of sections of gill tissue from (D) jack and (E–F) full-size coho salmon illustrate the measurements taken, where L is lamellae, GF is gill filament, LD is lamellae diameter, ED is erythrocyte diameter, and BWDD is blood-water diffusion distance.

Masses of the ventricle, gill basket and liver scaled close to isometrically with body mass (*b* = 1.05–1.07; Fig. 3). Spleen mass scaled slightly above isometric (*b* = 1.13±0.05). Gill histology revealed size-specific differences in gill filament diameter (*b* = 0.29±0.06), lamellae length (*b* = 0.34±0.12), and interlamellar distance (*b* = 0.19±0.02; Fig. 4). In contrast, the other gill histology measurements were independent of body mass: blood-water diffusion distance averaged 2.2±0.7 µm, gill lamellae diameter averaged 14.0±1.9 µm, and maximum erythrocyte diameter averaged 8.6±1.2 µm. Gonad mass scaled with an exponent of 0.84, indicating a relatively smaller gonad mass with increasing body mass and suggesting that jacks have a proportionately greater capacity for reproductive output (Fig. 3).

Following from the size-specific differences in plasma testosterone, potassium and osmolality detailed above, blood data from males were pooled for the 19 h post-exercise recovery group and the thermal challenge control group (both sampled at 7°C) to provide a large dataset (N = 38, *M*_b_ range 0.20–4.22 kg) to examine for any scaling of blood variables (Table 1). Significant relationships were found for plasma testosterone, potassium and osmolality, but not for any of the other measured blood parameters (Table 1). The same analyses performed on the male fish sampled at 20°C (N = 13) revealed no significant scaling between any blood variable and body mass, suggesting that the difference in plasma potassium detailed above (see *Thermal challenge*) was related to group differences (i.e., full-size males versus jacks) rather than body mass *per se*. Nevertheless, this finding should be taken with caution due to low sample size at 20°C (Table 1).

**Table 1:** Regression analyses describing the influence of body mass (*M*_b_) on three blood plasma variables of male coho salmon (*O. kisutch*) at different temperatures.

Plasma variable (*y*)	*a*	*b*	R^2^	F	P
*7°C (N = 38)*					
Testosterone	52.55±7.49	−0.510±0.139	0.328	17.56	0.0002
Potassium	2.33±0.21	−0.226±0.093	0.169	7.31	0.0104
Osmolality	299.25±0.86	0.009±0.003	0.184	8.13	0.0072
*20°C (N = 13)*					
Testosterone	17.30±4.15	−0.252±0.246	0.117	1.46	0.2519
Potassium	2.75±0.54	−0.070±0.218	0.010	0.11	0.7422
Osmolality	302.12±1.60	−0.001±0.006	0.001	0.007	0.9360

Equations are of the form *y* = *a* • *M*_b_ *^b^*.

## Discussion

### Body mass and thermal tolerance

There is a growing database for fishes to suggest that small adult individuals within a species may be more resilient to acute and chronic high temperature exposures than their larger conspecifics [1], [3], [5]. It is noteworthy that the only fish that did not survive the thermal challenge in the present study was the largest male. Significantly elevated plasma potassium in full-size males (including very high levels in the male that died) in comparison with jacks may point to a vital role of the ionoregulatory system in determining temperature tolerance. Additionally, size-specific differences in oxygen uptake across the gills may have played some role in the findings, as has been documented previously for Chinook salmon where large individuals maintained lower arterial oxygen content and subsequently had lower venous oxygen reserves to supply the spongy myocardium [5]. In contrast, most of the physiological and morphological variables measured in the present study either did not scale with body mass, or scaled isometrically, suggesting little contribution of these variables in determining size-specific thermal tolerance. The clear sex-specific differences in blood parameters in response to the temperature challenge (Fig. 1) help to explain the lower tolerance of female Pacific salmon to environmental perturbations [14], [29], [30].

While it has been proposed that the phenomenon of size-specific thermal tolerance in adult fishes is linked with a decrease in aerobic scope with increasing body mass (e.g., [3]), the results of the present study suggest that this is not the case in salmonids. Indeed, this is one of few studies to examine the intraspecific allometry of both minimum and active (peak) 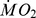 in mature fishes, and the results demonstrate that factorial aerobic scope changes little across body mass at 7°C (Fig. 3A). It should be noted that a similar exhaustive exercise protocol as the one used in this study elicited a higher maximum 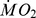 in Atlantic cod (*Gadus morhua*) than did a Ucrit test [31]. Furthermore, the values for 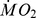
_peak_ in this study are only slightly lower than the values of maximum 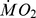 reported for full-size coho salmon exercising in a swim tunnel at the same temperature [24], and this difference could quite easily be associated with methodological rather than biological differences. Future research should investigate the thermal-dependence of the allometry of aerobic scope in adult fishes to determine whether this may help to explain size-specific thermal tolerance.

### Body mass and recovery from exercise

Despite the subtle size-specific responses of coho salmon to acute warming, clear size-specific differences in metabolic recovery from exhaustive exercise were apparent (Fig. 2A). Mass-specific EPOC was 28% less in jacks than full-size males and post-exercise recovery was 24% shorter. Interestingly in this regard, larger sockeye salmon (*O. nerka*) tended to have lower migration survival than smaller individuals when released in the marine environment *en route* to spawning grounds following a fisheries capture event [32]. Since ventricle mass scales isometrically with body mass in salmonids (Fig. 3C; [33]), it may be reasonable to assume that cardiac stroke volume also scales proportionally. While it was not possible to measure heart rate recovery in jacks in the present study, this is an obvious next step once appropriately-sized technology is developed. Nevertheless, resting heart rate is not dependent on body mass in mature Chinook salmon spanning a mass range of 2.7 to 16.8 kg [33], and cardiac output scales isometrically in Chinook salmon over the body mass range that has been examined to date (2.1–5.4 kg; [5], [33]). Others have demonstrated for a range of fish species that gill surface area generally scales with body mass with an exponent around 0.8 [34], [35], which is expectedly lower than the scaling exponent of 1.06 determined for the gill basket mass of coho salmon in the present study (Fig. 3D) since the former is a surface area (theoretically scales to body mass with *b* = ⅔) and the latter is a mass (theoretically scales to body mass with *b* = 1). Based on the above points, and the higher ventilation rate of jacks at 1 h post-exercise, it may seem reasonable to assume that metabolic recovery of full-size fish may have been limited by oxygen uptake at the gills rather than by convective oxygen transport through the circulatory system. However, isometric scaling of 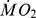
_peak_, constant blood-water diffusion distance across body mass, and expected scaling (i.e., *b*∼⅓) of gill filament diameter (*b* = 0.29) and lamellae length (*b* = 0.34) all suggest that there was no size-specific effect on the rate of either diffusive or convective oxygen transport.

A critic could argue that jacks did not exhaust themselves as much as full-size fish during the exercise protocol, which subsequently caused the size-specific differences in metabolic recovery. However, this is highly unlikely since jacks appeared equally exhausted as full-size fish during and following the protocol, and plasma lactate and cortisol reached similar levels independent of fish size (Fig. 2). Indeed, there was little evidence for an attenuated blood stress response or a faster recovery of plasma lactate and cortisol that allowed jacks to achieve a faster metabolic recovery (also see [36], [37]). Instead, it seems likely that larger fish simply had a greater ‘oxygen debt’ that took longer to pay back at a size-independent 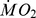
_peak_ of ∼6 mg min^−1^ kg^−1^. Regaining osmoregulatory homeostasis is a contributor to the oxygen debt recovery and jacks appeared to accomplish this faster than full-size males following the exhaustive exercise protocol (Fig. 2I). Previously, it was shown that exercise-trained juvenile Chinook salmon were better able to maintain osmoregulatory status during and following a prolonged swimming challenge to exhaustion when compared with similarly-sized untrained fish [38]. Whether such effects of training and body size are related to an improved ability to manage the osmo-respiratory compromise [39], [40] or some other process is unclear.

Adding to the known effects of stress on reproductive maturation and gamete quality (e.g., [41]), the present study discovered a novel size effect for hormonal recovery in the form of a delay in restoration of plasma testosterone in full-size males (Fig. 2K). Maturing salmonids elevate plasma testosterone as part of gonadal development [42], and so it is possible that the maturation trajectory of jacks may be less impaired than full-size males following an exhaustive interaction with a fishery or an animal predator. Since the strategy of returning as a jack has a genetic component in coho salmon [43], [44], the possibility exists for selection towards this life history trait if selection pressure is sufficient.

### Conclusions

Fisheries have been implicated in modifying the size structure of fish populations by harvesting larger individuals and avoiding or releasing smaller ones [21], [45], [46], [47], [48], [49]. More recent data suggest that, independent of fisheries, the warming global climate is compounding this issue by selecting for smaller fish species and smaller individuals within a species [1], [3]. Despite an extensive investigation, this study did not discover any obvious physiological mechanisms or morphological attributes that may underlie superior thermal tolerance in small individuals, although disturbance to ionoregulatory homeostasis emerged as one of the more likely factors. Nevertheless, the present study highlighted yet another issue in the form of size-specific recovery of homeostasis (e.g., metabolic, osmoregulatory and hormonal recovery) following exhaustive exercise that may have a proportionately more negative effect on larger than smaller conspecifics when released following capture. With multiple drivers selecting for smaller individuals, it seems likely that average fish mass and size at maturity will continue to decrease for many species, with those species interacting with fisheries likely to experience the greatest impact. Given the substantial ecological and economical implications of this issue, particularly with a rapidly increasing human reliance on fish products, further research is imperative to help understand the underlying mechanisms and guide sustainable fishing practices in a changing world.
